# *Slc25a5* regulates adipogenesis by modulating ERK signaling in OP9 cells

**DOI:** 10.1186/s11658-022-00314-y

**Published:** 2022-02-02

**Authors:** Shenglong Zhu, Wei Wang, Jingwei Zhang, Siyu Ji, Zhe Jing, Yong Q. Chen

**Affiliations:** 1 Wuxi School of Medicine, Jiangnan University, 1800 Lihu Road, Wuxi, 214122 Jiangsu China; 2 Wuxi Translational Medicine Research Center and Jiangsu Translational Medicine Research Institute Wuxi Branch, Wuxi, China; 3 School of Food Science and Technology, Jiangnan University, Wuxi, China

**Keywords:** Obesity, Adipogenic differentiation, *Slc25a5*, ERK, Transcriptome, Metabolome

## Abstract

**Background:**

A comprehensive understanding of the molecular mechanisms of adipogenesis is a critically important strategy for identifying new targets for obesity intervention.

**Methods:**

Transcriptomic and lipidomic approaches were used to explore the functional genes regulating adipogenic differentiation and their potential mechanism of action in OP9 cells and adipose-derived stem cells. Oil Red O staining was used to detect oil droplets in adipocytes.

**Results:**

RNA sequencing (RNA-seq) showed that *Slc25a5* expression was significantly upregulated in adipogenic differentiation. Depletion of *Slc25a5* led to the suppressed expression of adipogenesis-related genes, reduced the accumulation of triglycerides, and inhibited PPARγ protein expression. Moreover, the knockdown of *Slc25a5* resulted in significant reduction of oxidative phosphorylation (OXPHOS) protein expression (ATP5A1, CQCRC2, and MTCO1) and ATP production. The RNA-seq and real-time quantitative polymerase chain reaction (RT–qPCR) results suggested that adipogenic differentiation is possibly mediated by ERK1/2 phosphorylation, and this hypothesis was confirmed by intervention with PD98059 (an ERK 1/2 inhibitor).

**Conclusions:**

This study indicates that *Slc25a5* inhibits adipogenesis and might be a new therapeutic target for the treatment of obesity.

**Supplementary Information:**

The online version contains supplementary material available at 10.1186/s11658-022-00314-y.

## Background

The incidence of obesity, defined by abnormal or excessive fat accumulation, is increasing worldwide, with 13% of adults being obese [1]. Obesity is a significant risk factor for several metabolic syndromes, including cardiovascular disease, type 2 diabetes, nonalcoholic fatty liver disease, hypertension, and cancer [2]. Hyperplasia and hypertrophy of adipocytes are major contributors to obesity. These two processes are largely dependent on the regulation of adipogenesis [3]. Adipogenesis is a complicated process by which preadipocytes differentiate into mature adipocytes, and it is mediated by a series of key transcription factors, including γ-cytidine–cytidine–adenosine–adenosine–thymidine (CCAAT)/enhancer binding protein (c/EBP)-α and peroxisome proliferator-activated receptor (PPAR)-γ [4]. PPARγ and C/EBPα regulate each other, and drive the expression of genes involved in lipogenesis, lipolysis, and insulin sensitivity, such as fatty acid synthase (*FASN*), fatty acid-binding protein (*FABP4*), glucose transporter 4 (*GLUT4*), and lipoprotein lipase (*LPL*) [5–7]. A better understanding of adipogenesis is important to human health. However, the adipogenic differentiation process involves numerous pathways, and many potential targets for suppressing fat synthesis have yet to be discovered.

Thus, to discover potential factors involved in regulating adipogenesis, we utilized high-throughput sequencing to identify known genes through two classical adipogenic differentiation models and identified solute carrier family 25-member 5 (*Slc25a5*) gene as a potentially key gene regulating adipogenic differentiation that might be a new therapeutic target for the treatment of obesity.

## Methods

### Reagents

Dulbecco’s modified Eagle medium/nutrient mixture F-12 (DMEM/F12; Thermo Fisher Scientific, 11330032), Minimum Essential Medium α (αMEM; Thermo Fisher Scientific, 12561056), jetPRIME transfection reagent (Polyplus-transfection, 114-15), fetal bovine serum (FBS; VivaCell Biotechnology, C04001), rosiglitazone (MedChemExpress, HY-17386), insulin (Shanghai Yuanye Bio-Technology, S12033), 3-isobutyl-1-methylxanthine (IBMX; Aladdin Biochemical Technology, I106812), type I collagenase (Sangon Biotech, A004194), penicillin G sodium salt (Sangon Biotech, A600135), streptomycin sulfate (Sangon Biotech, A610494), epidermal growth factor (EGF; GenScript, Z02972), basic fibroblast growth factor (bFGF; GenScript Biotech, Z03016), GlutaMAX (Thermo Fisher Scientific, 35050061), trypsin (Sangon Biotech, A100458), Oil Red O (Sangon Biotech, A600395), Ultrapure RNA Kit (CWBIO, CW0581M), triglyceride (TG) assay kit (Nanjing Jiancheng Bioengineering Institute, A110-1-1), PD98059 (MedChemExpress, HY-12028), radioimmunoprecipitation assay (RIPA) buffer (Beyotime Biotechnology, P0013B), polyvinylidene fluoride (PVDF) membranes (Millipore, ISEQ00010), Enhanced chemiluminescent reagent (ECL) (Millipore, WBKLS0500), ATP assay kit (Beyotime Biotechnology, S0026), Hieff UNICON qPCR SYBR green master mix (Shanghai YEASEN Biotech, 11198ES), total protein assay kit (Nanjing Jiancheng Bioengineering Institute, A045-4-2), HiFi PCR mix for next-generation sequencing (NGS) (CWBIO, CW2648), HiScript III first-strand cDNA synthesis kit (Vazyme Biotech, R312-02) and second-strand cDNA synthesis kit (Beyotime Biotechnology, D7172), Extracellular signal-regulated kinase (ERK) (Cell Signaling Technology, 4695), p-ERK (Cell Signaling Technology, 4370), C-Jun N-Terminal Kinase (JNK) (Cell Signaling Technology, 9252), p-JNK (Cell Signaling Technology, 4668), P38 (Cell Signaling Technology, 8690), p-P38 (Cell Signaling Technology, 4511), anti-slc25a5 (Biodragon Immunotech, BD-PN0419), anti-β-actin (ABclonal, AC026), anti-MTCO1 (ABclonal, A17889), anti-SDHB (Proteintech, 10620-1-AP), anti-ATP5A1 (Proteintech, 14676-1-AP), anti-UQCRC2 (Proteintech, 14742-1-AP), and anti-NDUFB8 (Proteintech, 14794-1-AP). Tn5 transposase was purified according to a published protocol [8].

### Cells and treatments

Mouse OP9 cells (CCTCC, Wuhan, China) were cultured in MEM supplemented with 5% fetal bovine serum (FBS) and 1% penicillin–streptomycin at 37 °C and 5% CO_2_. To induce differentiation, 100% confluent OP9 preadipocytes were stimulated with 1 mM rosiglitazone in DMEM containing 5% FBS for 10 days. Mouse adipose-derived stem cells (ASCs) were isolated following previously described methods [9]. Briefly, 8–10-week-old male C57/BL6J mice were sacrificed and subcutaneous adipose tissue taken from the inguinal fat pads. The subcutaneous adipose tissue was washed, minced, and digested. Finally, the pellet was collected and filtered through a 70 μm cell strainer. The cells were cultured in growth medium (DMEM/F12 containing 5 ng/ml EGF, 5 ng/ml bFGF, and GlutaMAX). Next, cells were grown to 90% confluence and then changed to adipogenic differentiation medium (DMEM supplemented with 5% FBS and 1 μM rosiglitazone) for 10 days.

### RNA interference

Mouse OP9 cells were grown to 50–70% confluence and then transfected with *Slc25a5* small interfering RNA (siRNA) (10 nM) or negative control (NC) siRNA (10 nM) using jetPRIME transfection reagent according to the manufacturer’s protocol. Cells were incubated for 24 h prior to MEM being replaced with adipogenic differentiation medium. The following siRNAs specific to *Slc25a5* were designed by Tianlin Biotechnology (Table 1). Negative control siRNA (GenePharma, A06001) was used as a control.

**Table 1:** Sequences for siRNA

siRNAs	sense (5′–3′)	antisense (5′–3′)	Organisms
siRNA1	GCCUUUGUGCUUGUCUUGUAUTT	AUACAAGACAAGCACAAAGGCTT	Mouse
siRNA2	GCUGCCUACUUUGGUAUCUAUTT	AUAGAUACCAAAGUAGGCAGCTT	Mouse
siRNA3	GCAAGCAAAUCACGGCAGAUATT	UAUCUGCCGUGAUUUGCUUGCTT	Mouse
siRNA4	GGUAUCUAUGACACUGCAATT	UUGCAGUGUCAUAGAUACCTT	Mouse
siRNA5	GCUCCCAGAUCCCAAGAAUTT	AUUCUUGGGAUCUGGGAGCTT	Mouse

### Oil Red O staining

Differentiated cells were washed with phosphate-buffered saline (PBS), fixed in 4% paraformaldehyde for 30 min, and then washed three times with PBS. The cells were stained with 60% saturated Oil Red O for 15 min. After staining, the cells were washed with 60% isopropanol in PBS and then counterstained with hematoxylin solution.

### ATP measurement

ATP content was measured using the ATP assay kit following the manufacturer’s instructions.

### RNA extraction and RT–qPCR assay

Total RNA was extracted from cells using an Ultrapure RNA Kit following the manufacturer’s protocol, after which cDNA was prepared with a HiScript III first-strand cDNA synthesis kit. Hieff UNICON qPCR 109 SYBR Green Master Mix was used for qRT–PCR, which was performed with a Roche LightCycler 480 PCR System. Relative mRNA expression levels were evaluated using the 2^−ΔΔCt^ method and normalized to the GAPDH mRNA expression level. The primers are described in Table 2.

**Table 2:** Primers for RT-qPCR

Gene	Forward (5′–3′)	Reverse (5′–3′)	Organisms
*GAPDH*	AGGTCGGTGTGAACGGATTTG	TGTAGACCATGTAGTTGAGGTCA	Mouse
*FABP4*	AAGGTGAAGAGCATCATAACCCT	TCACGCCTTTCATAACACATTCC	Mouse
*C/EBPα*	GCGGGAACGCAACAACATC	GTCACTGGTCAACTCCAGCAC	Mouse
*FASN*	AGAGATCCCGAGACGCTTCT	GCTTGGTCCTTTGAAGTCGAAGA	Mouse
*PPARγ*	TCGCTGATGCACTGCCTATG	GAGAGGTCCACAGAGCTGATT	Mouse
*Slc25a5*	CAAGACAGCGGTAGCACCC	CGCAGTCTATGATGCCCTTGTA	Mouse

### Measurement of triglyceride (TG)

The TG content of the cells was measured as previously described [10, 11]. TG content was normalized to total cellular protein.

### Western blot

Total protein was collected from cells using RIPA buffer, and western blotting was performed as previously described [12]. Briefly, the protein (50 μg) was separated by 10% sodium dodecyl sulfate (SDS)–polyacrylamide gel electrophoresis and transferred to a PVDF membrane. The membrane was blocked with 5% defatted milk and incubated with specific primary antibodies against PPARα, ERK, p-ERK, JNK, p-JNK, P38, p-P38, β-actin, slc25a5, ATP5A1, SDHB, UQCRC2, MTCO1, and NDUFB8 at 4 °C overnight. Finally, the membrane was incubated with a secondary antibody, anti-rabbit IgG conjugated to horseradish peroxidase (HRP), at room temperature for 2 h. Immunodetection was visualized using an enhanced chemiluminescent (ECL) reagent (Biotanon Biotechnology, Shanghai, China). Quantification of band intensity was performed using ImageJ software (version 1.53; National Institutes of Health).

### Lipidomic analysis and fatty acid methyl ester (FAME) analysis

Lipids were extracted by two-phase separation following a previously described method [13]. Lipidomic data were analyzed with an Exactive Plus mass spectrometer (Thermo Scientific) equipped with a Vanquis UHPLC (Thermo Scientific) and operated in positive and negative ion modes. Lipids were eluted on an ACQUITY UPLC BEH C18 column (1.7 μm, 2.1 × 100, Waters) at 40 °C with a gradient of mobile phases A (10 mM ammonium acetate in acetonitrile: H_2_O) and B (10 mM ammonium acetate in isopropyl alcohol:acetonitrile). A quality control (QC) spike mixture was prepared by mixing different samples to investigate the interrun and interproject effects. Lipid annotation was achieved using LipidMap (www.lipidmaps.org), and statistical analysis was performed using SIMCA (14.1) software. FAME analysis was performed as described previously [14]. C15:0 was used to normalize the results.

### RNA sequencing (RNA-seq)

A total of 1 μg of RNA per sample was used to construct sequencing libraries. Briefly, the RNA was converted into double-stranded cDNA following the reverse-transcription kit manufacturer’s recommendations, and then double-stranded cDNA was digested and labeled by using Tn5 transposase. Finally, enrichment PCR was performed using HiFi PCR Mix for NGS. The libraries were then quantified using an Agilent 2100 Bioanalyzer. Paired-end sequencing of the library was performed on an Illumina NovaSeq instrument (sequencing was performed by GENEWIZ Biotech). Reads were mapped to the mouse genome using STAR (http://code.google.com/p/rna-star/). Genes showing a ≥ 1.5-fold change (*P* < 0.05) were considered to be significantly differentially expressed. Gene Ontology (GO) enrichment analysis of differentially expressed genes (DEGs) was performed using Metascape (http://metascape.org).

### Statistical analysis

The data are presented as the mean ± standard error of the mean (SEM). All experiments were repeated at least three times. GraphPad Prism 6.0, SPSS 19.0, and R 3.6.0 were used for data analyses. Student’s *t*-tests and one-way analysis of variance were used to analyze the differences between the groups. *P* < 0.05 was regarded as statistically significant.

## Results

### *Slc25a5* is increased in adipogenic differentiation

**Fig. 1:**
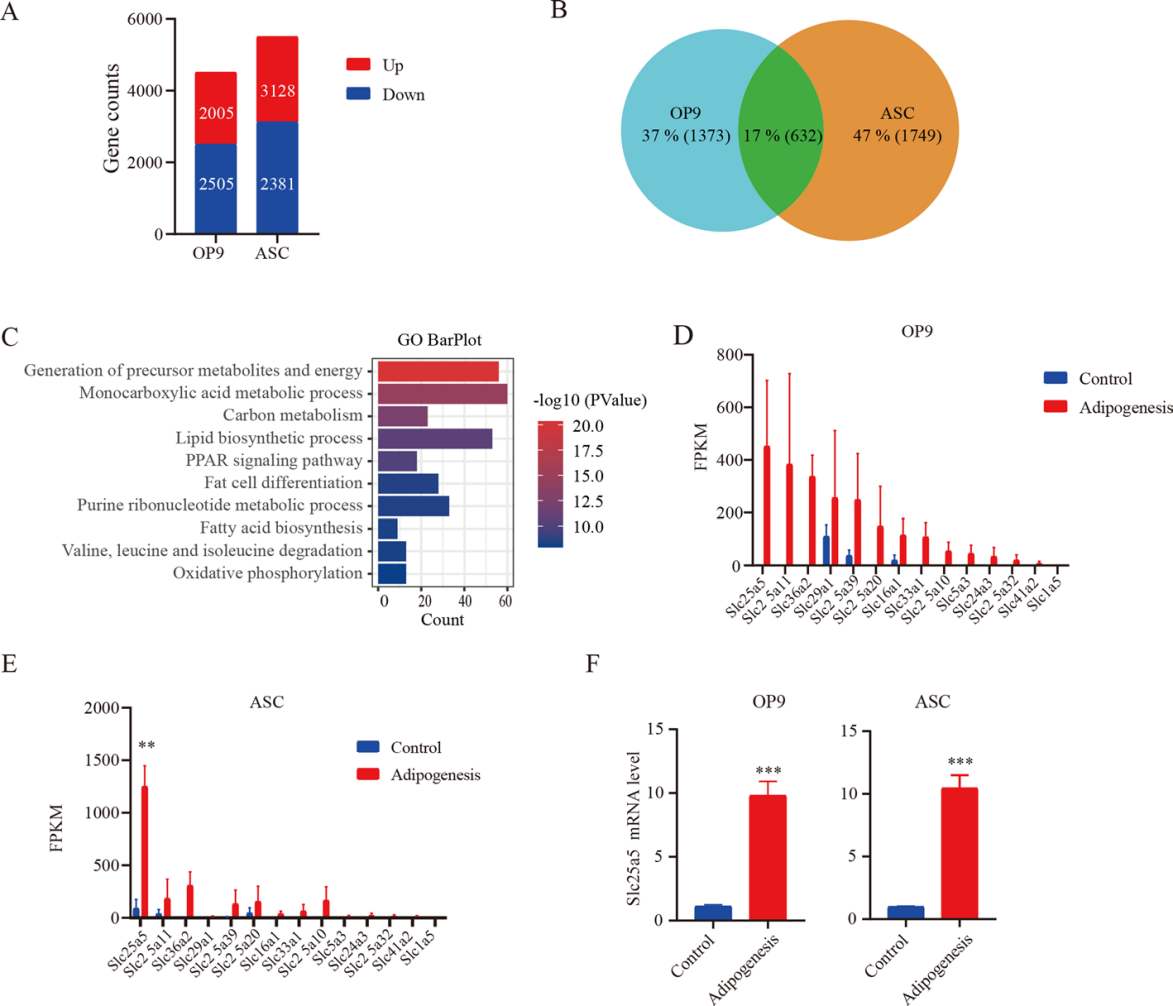
*Slc25a5* is increased in adipogenic differentiation. RNA-seq data were obtained from OP9 cells and ASCs undergoing adipogenic differentiation and those that were not. **A** Upregulated and downregulated gene expression profiles were selected. **B** Venn diagram illustrating the upregulated genes in both OP9 and ACSs during adipogenic differentiation. **C** Ten GO terms representing the most upregulated genes during adipogenic differentiation. **D**, **E** Expression profiles of the *Slc25* gene family during adipogenic differentiation. **F** The relative mRNA level of *Slc25a5* was measured with RT–qPCR. The values are mean ± SEM (*n* = 3). **P < 0.01; ***P < 0.001 versus controls

As shown in Additional file 1: Fig. S1, the differentiation of adipogenic differentiation medium-treated OP9 and ASC cells was successful as evidenced by Oil Red O staining and RT–qPCR. To determine the key genes that are involved in adipogenic differentiation, the transcriptome was assessed. RNA-seq data were obtained from OP9 and ASCs that differentiated into adipose cells and those that were undifferentiated. As shown in Fig. 1A, 2005 and 2381 genes were upregulated during adipogenic differentiation of OP9 and ASC (fold change > 2), respectively. Further analysis revealed 632 genes that were upregulated in both cell lines (Fig. 1B). To perform a gene functional annotation and pathway enrichment assay, Metascape was used to perform GO analysis. The ten GO terms enriched with the greatest number of DEGs among the 632 genes identified are shown in Fig. 1C. This result indicated that adipogenic differentiation was achieved through mitochondrial oxidative metabolism. Among the most differentially expressed genes that had not been previously studied in adipogenic differentiation, we focused on *Slc25* family genes, which were significantly upregulated. The *Slc25* family is a mitochondrial ADP/ATP carrier that imports ADP into the mitochondrial matrix and provides metabolism-produced energy for cell survival by exporting newly synthesized ATP to the cytosol. This GO analysis finding may hint at a new role for the *Slc25* family. Further analysis showed that 14 genes in the *Slc25* family were upregulated during adipogenic differentiation. Of these, *Slc25a5* was the most significantly upregulated in the two classical adipogenic differentiation models (Fig. 1D, E). Furthermore, qPCR confirmed that the mRNA level of *Slc25a5* was increased during adipogenesis (Fig. 1F). These results suggest that *Slc25a5* participates in adipogenic differentiation.

### Inhibiting *Slc25a5* prevents adipogenic differentiation

**Fig. 2:**
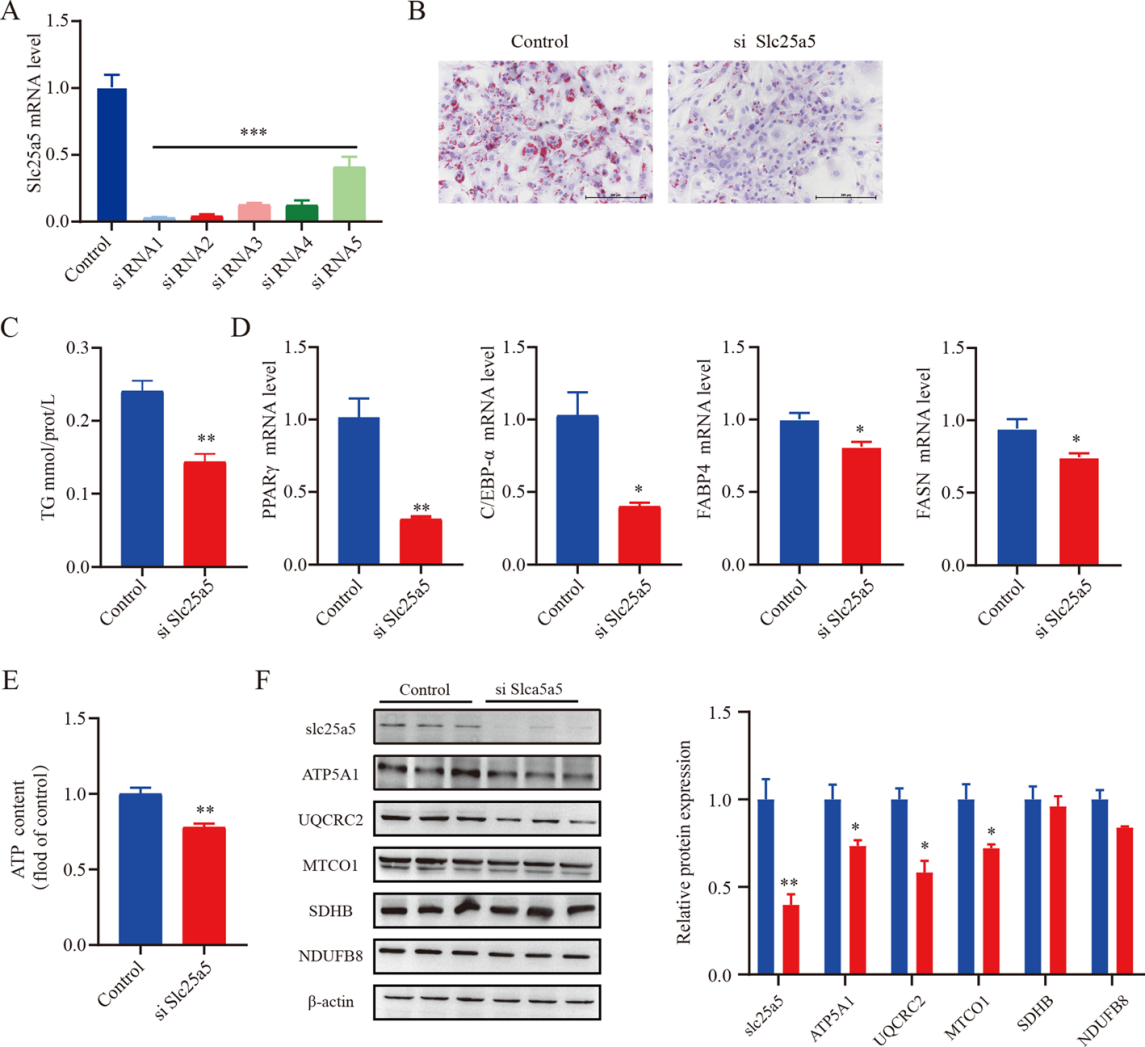
Inhibiting *Slc25a5* prevents adipogenic differentiation. Before adipogenic differentiation, OP9 cells were transfected with *Slc25a5* siRNA, and 24 h later, the cells were incubated with 1 mM rosiglitazone to induce adipogenic differentiation. **A** RT–qPCR verified the efficiency of *Slc25a5* knockdown in OP9 cells. **B** Oil Red O staining; scale bar is 200 μm. **C** Cellular TG levels were measured. **D** RT–qPCR was performed to assess the expression of the adipogenic factors PPARγ, FABP4, FASN, and C/EBPα. **E** Western blot of selected subunits of the OXPHOS complexes. **F** Cellular levels of ATP. Values are the mean ± SEM (*n* = 3). *P < 0.05; **P < 0.01 versus control

OP9 cells have the advantages of rapid adipogenic differentiation and high transfection efficiency. Because of these attributes, OP9 cells were used in the following experiments. To classify the role of *Slc25a5* in adipocyte differentiation, five siRNAs were designed to silence *Slc25a5* during adipogenic differentiation. Compared with cells treated with control siRNA, the cells transfected with siRNA1 showed a 90% reduction in the level of *Slc25a5*, as determined by qPCR analysis (Fig. 2A). siRNA1 was used in the following studies. Our results revealed that *Slc25a5* knockdown significantly decreased the number of lipid droplets and the level of TG accumulation (Fig. 2B, C). In addition, expression of the adipocyte differentiation marker genes *PPARγ*, *FABP4*, *FASN*, and *C/EBPα* was decreased dramatically in *Slc25a5*-silenced cells (Fig. 2D). The same results were also observed in another siRNA2 (Additional file 2: Fig. S2). These results suggest that *Slc25a5* plays a crucial role in adipogenesis. Mitochondria synthesize ATP through five complexes controlling OXPHOS. Western blot of OXPHOS complexes revealed lower levels of these proteins (ATP5A1, CQCRC2, and MTCO1) in *Slc25a5*-silenced cells (Fig. 2E), suggesting that *Slc25a5* silencing suppressed oxidative capacity of the mitochondria. Meanwhile, cellular ATP level was markedly reduced in *Slc25a5*-silenced cells (Fig. 2F).

### *Slc25a5* silencing decreases the content of cellular fatty acids

**Fig. 3:**
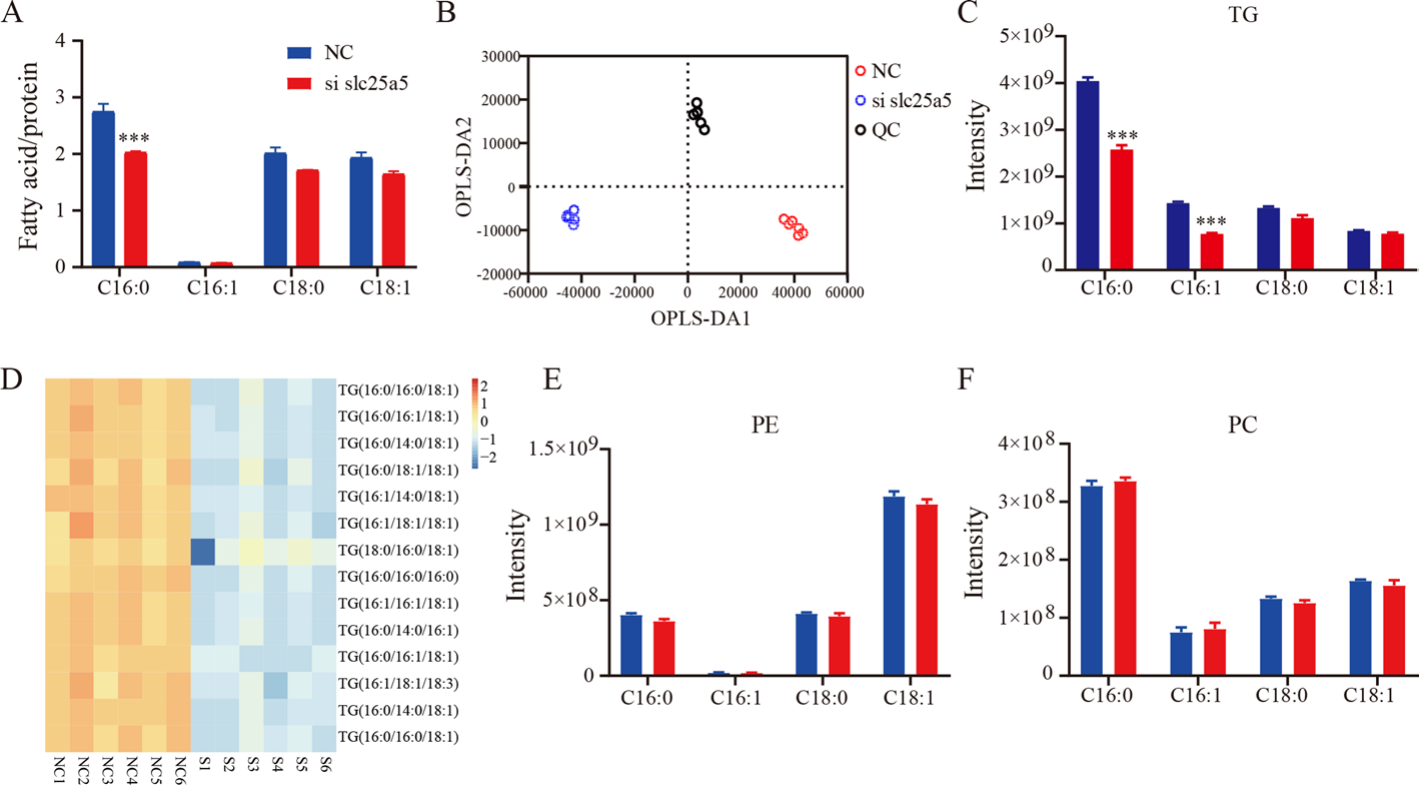
*Slc25a5* silencing decreases the content of cellular fatty acids. **A** Cellular fatty acids were identified by GC–MS after adipogenic differentiation. **B** OPLS-DA score plot showing clear clustering between the NC and *Slc25a5*-silenced groups. **C** The composition of TGs (C16:0, C16:1, C18:0, and C18:1) among lipids. **D** The differential lipid composition of TGs was determined by VIP scores (VIP > 2, *P* < 0.05). **E**, **F** The proportion of PE and PC (C16:0, C16:1, C18:0, and C18:1) among lipids. The values are the mean ± SEM (*n* = 6). ***P < 0.001 versus control

To identify the types of fatty acids that are decreased in the cells with *Slc25a5* knocked down after adipogenic differentiation, the fatty acid content of the cells with or without *Slc25a5* knockdown after adipogenic differentiation was measured by gas chromatography–mass spectrometry (GC–MS). Compared with that in control cells, the content of C16:0 was significantly decreased in *Slc25a5*-knockdown cells (the levels of other fatty acids were not markedly different, as shown in Fig. 3A). To further characterize the lipid composition, liquid chromatography (LC)–MS-based lipidomics were assessed to determine the types of lipids that were decreased in *Slc25a5*-knockdown cells. An orthogonal partial least-squares discrimination analysis (OPLS-DA) supervised model was established to compare the lipid changes between the NC, *Slc25a5* knockdown, and QC groups. The OPLS-DA revealed clear distinct clustering between the NC and *Slc25a5*-silenced groups (Fig. 3B). In addition, compared with that in the control, in the cells with silenced *Slc25a5*, there was a significant decrease in the content of TG, especially the content of C16:0 and C16:1 (Fig. 3C), but not in phosphatidylethanolamine (PE) or in phosphatidylcholine (PC) (Fig. 3E, F). The abundance of 14 triglycerides was significantly decreased in *Slc25a5*-silenced cells (*P* < 0.05 and Variable importance in the projection (VIP) > 2) (Fig. 3D). This finding suggests that *Slc25a5* deficiency mainly influences TG biosynthesis.

### Transcriptome analysis implicates the ERK pathway as a target of *Slc25a5*

**Fig. 4:**
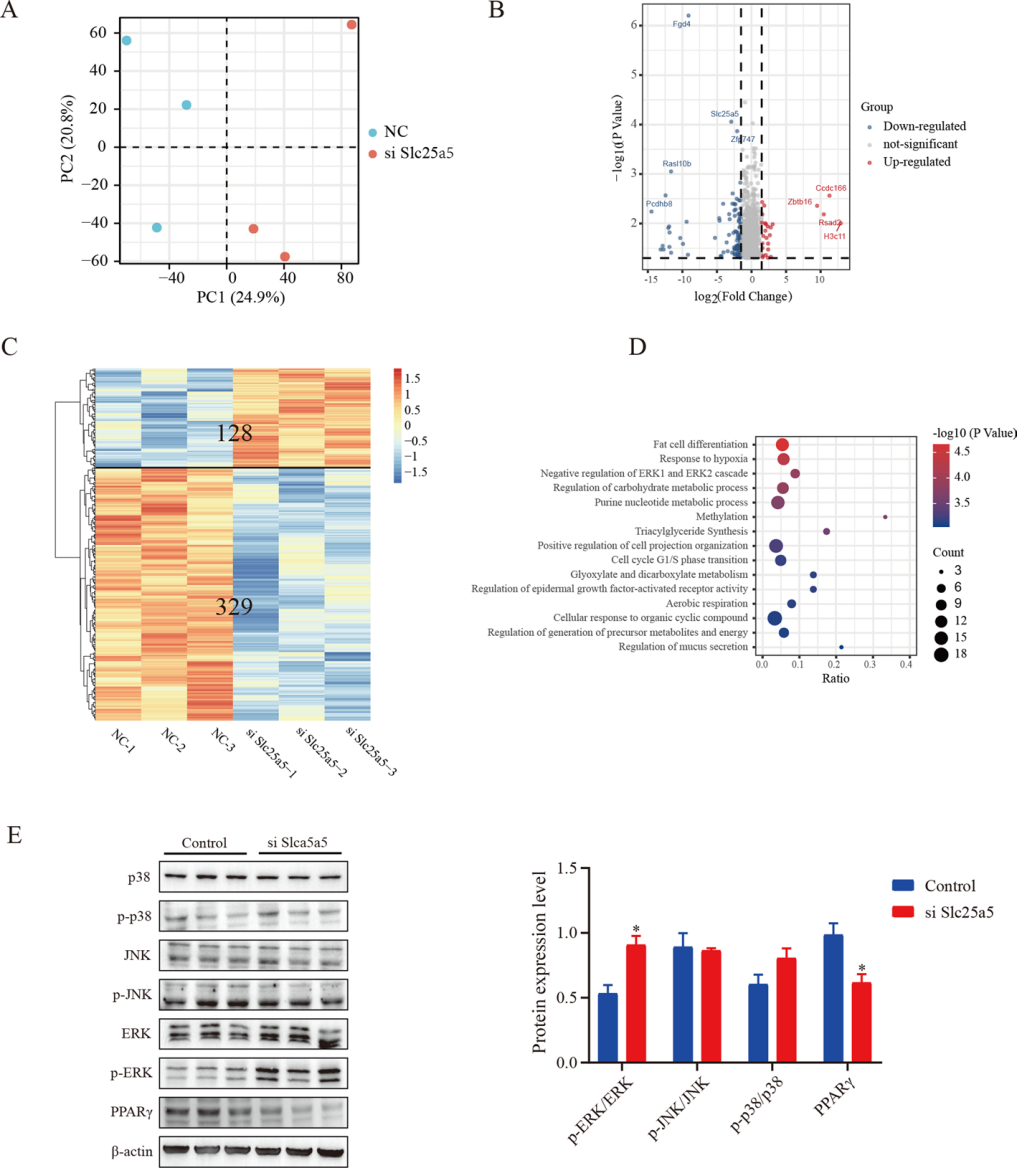
Transcriptomics analysis implicates ERK pathway as a target of *Slc25a5*. After adipogenic differentiation, OP9 cells with or without *Slc25a5* knockdown were collected, and a transcriptome analysis was performed. **A** PCA score plot showing clear clusters in each group. **B** Scatterplot of DEGs between the control and *Slc25a5*-knockdown cells (fold change ≥ 1.5, and *P* ≤ 0.06). **C** Heat map of the DEGs between the control and *Slc25a5*-knockdown cells. **D** GO analysis of downregulated DEGs. **E** Protein levels of ERK, p-ERK, P38, p-P38, JNK, p-JNK, and PPARγ were measured by western blot analysis. The values are the mean ± SEM (*n* = 3). *P < 0.05 versus control

To explore the underlying mechanism of *Slc25a5* in adipogenesis, the gene expression profiles with or without *Slc25a5* knockdown were identified by RNA sequencing. Principal component analysis (PCA) was used to distinguish each experimental group, and the PCA results showed that each group was completely separated (Fig. 4A). Genes with a fold change ≥ 1.5 and *P* < 0.05 were defined as DEGs. Based on these criteria, a total of 457 DEGs were identified, including 329 downregulated genes and 128 upregulated genes (Fig. 4B, C). A GO analysis was performed to investigate the potential functions of the downregulated genes. Significantly enriched functional categories included fat cell differentiation, response to hypoxia, chemokine signaling, negative regulation of the ERK1 and ERK2 cascade, and triglyceride synthesis (Fig. 4D). However, no specifically enriched biological processes were found among the upregulated genes. Previous studies have demonstrated that *Slc25a5* deficiency improves obesity-induced adipocyte hypoxia and insulin resistance [15]. In addition, ERK signaling is necessary to initiate the adipogenic differentiation process [16]. Hence, we speculated that the ERK signal was involved in the blockage of adipocyte differentiation induced by *Slc25a5* deficiency. The western blot results confirmed our hypothesis. As shown in Fig. 4E, *Slc25a5* knockdown significantly induced ERK1/2 phosphorylation during adipocyte differentiation, but other MAPKs (JNK and p38) were not changed. Previous studies have indicated that the activation of ERK1/2 can suppress the transcriptional activity of PPARγ, thereby inhibiting adipocyte differentiation [17]. Consistently, our results showed that the protein level of PPARγ was significantly decreased in the *Slc25a5*-deficient group (Fig. 4E).

### An ERK inhibitor reverses *Slc25a5* knockdown-induced adipogenesis inhibition

**Fig. 5:**
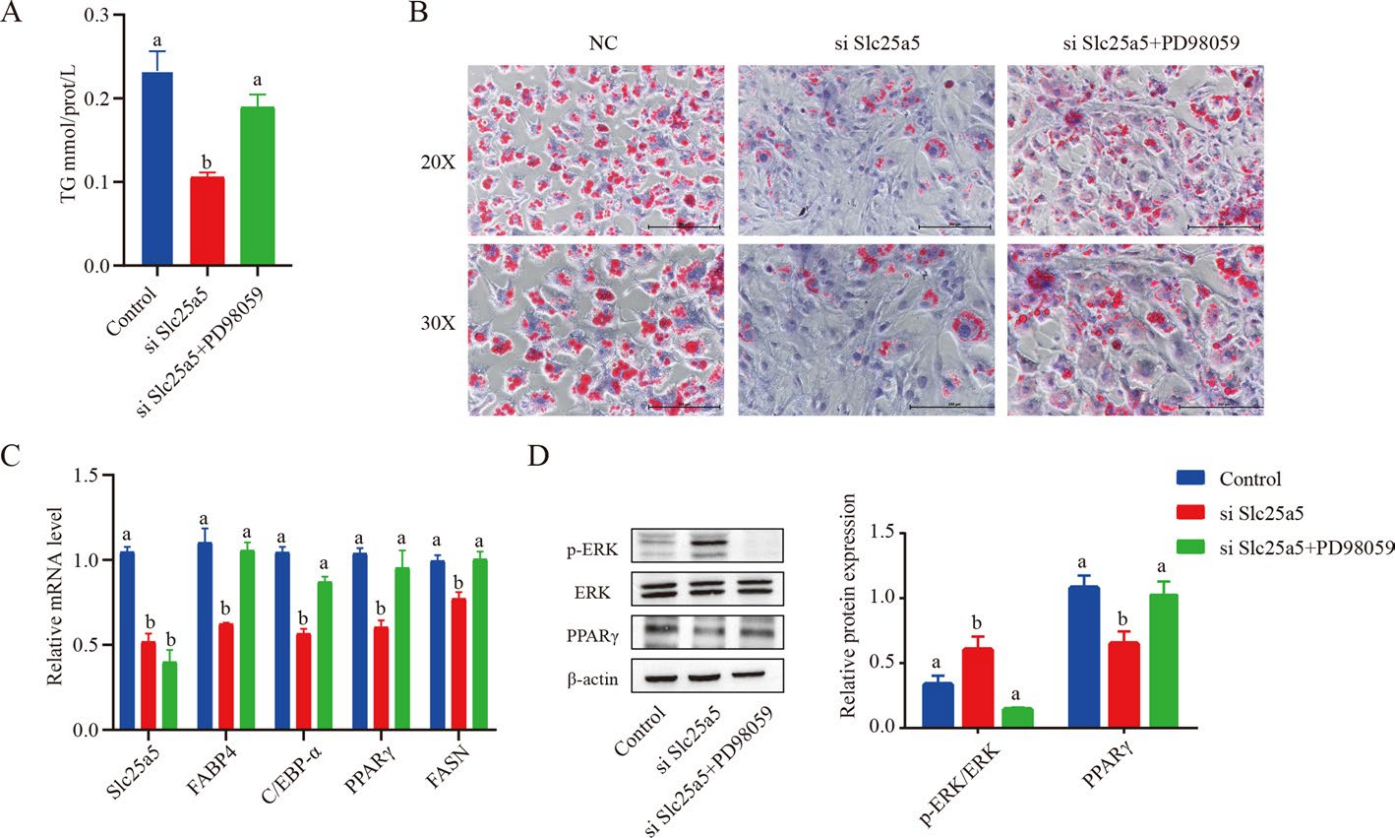
ERK inhibitor reverses *Slc25a5* knockdown-induced adipogenesis inhibition. Before adipogenic differentiation was induced, OP9 cells were transfected with *Slc25a5* siRNA, and 24 h later, these cells were incubated with 1 mM rosiglitazone and PD98059 (50 μM). **A** Cellular triglyceride (TG) levels were measured. **B** Oil Red O staining was performed after adipogenic differentiation, and the results were visualized by light microscopy. The scale bar is 200 µm. **C** RT–qPCR was performed to assess the expression of the adipogenic factors *PPARγ*, *FABP4*, *FASN*, and *C/EBPα* after adipogenic differentiation. **D** Protein levels of ERK, p-ERK, and PPARγ were measured by western blot analysis. The values are the mean ± SEM (*n* = 3). a versus b represents differences between groups; b versus b represents no differences between groups

To verify that *Slc25a5* regulates adipogenesis in an ERK signaling-dependent manner, the effect of a specific ERK1/2 inhibitor (PD98059) was assessed. PD98059 significantly reversed the effects of *Slc25a5* knockdown-induced adipogenesis inhibition, and the number of lipid droplets and TG level were markedly increased in *Slc25a5*-silenced cells treated with PD98059 (Fig. 5A, B). qPCR analysis further demonstrated that ERK inhibition blocked the downregulation of adipogenic genes due to *Slc25a5* knockdown (*PPARγ, FABP4, FASN*, and *C/EBPα*) (Fig. 5C). These results suggested that *Slc25a5* regulates adipogenic differentiation by mediating the ERK pathway.

## Discussion

Preadipocytes undergo coordinated and multifaceted changes in gene expression and protein content as they differentiate into mature adipocytes. Understanding the molecular events regulating adipogenesis may lead to an effective treatment of obesity.

How can potential biomarkers be screened quickly and efficiently? To answer this question, we performed RNA-seq with preadipocytes and adipocytes in two classical adipogenic models. The RNA-seq analysis led to the identification of 632 genes that were upregulated in both OP9 and ASC cells. Through a bioinformatics analysis, *Slc25a5* was identified as a gene expected to be critically involved in adipogenesis. *Slc25a5*, also known as ANT2, facilitates the exchange of ADP and ATP between the mitochondria and the cytoplasm [18]. Previous studies have suggested that oxidative phosphorylation is essential for normal adipogenic differentiation, as ATP fuels normal adipogenesis during the initial step of adipogenesis. An increase in ATP production contributes not only to adipogenesis but also to excessive lipid accumulation-induced obesity [19]. In our study, *Slc25a5* was found to be significantly increased during adipogenic differentiation, and *Slc25a5* silencing suppressed the oxidative capacity of the mitochondria. This led to insufficient cytosolic ATP, resulting in the inhibition of adipogenic differentiation.

We next explored the molecular mechanism by which *Slc25a5* regulates adipocyte differentiation. RNA-seq was used to reconstruct de novo transcriptomes of *Slc25a5* silencing, and ERK signaling was found to be involved in adipogenesis. The MAPK/ERK signaling pathway has been intensively investigated in adipogenesis because it regulates a variety of transcription factors involved in adipocyte growth and differentiation [16, 20]. In addition, previous studies have suggested that ERK phosphorylation is necessary for the expression of PPARγ and that sustaining ERK phosphorylation can downregulate PPARγ expression [21, 22]. Our results indicated that *Slc25a5* silencing significantly increased ERK1/2 phosphorylation and suppressed the protein expression of PPARγ during adipocyte differentiation. These results further confirmed that *Slc25a5* silencing can suppress adipogenesis by regulating the protein stability of PPARγ. Numerous studies have reported that key adipogenic genes, such as *PPARγ*, *FABP4*, *FASN*, and *C/EBPα*, directly participate in adipogenic differentiation [23, 24]. PPARγ and C/EBPα together promote differentiation by activating adipose-specific gene expression, such as *FABP4* and *FASN*, and by maintaining each other’s expression at high levels [25]. *Slc25a5* knockdown markedly decreased the expression of adipogenic marker genes, indicating that the *Slc25a5*-ERK pathway is an upstream regulator of adipogenic differentiation. Therefore, the *Slc25a5*-ERK pathway can be a key therapeutic target for the management of obesity.

## Conclusions

In conclusion, we identify *Slc25a5* as a novel and important regulator in adipocyte differentiation. *Slc25a5* is highly expressed during adipogenesis, and knockdown of *Slc25a5* inhibits adipocyte differentiation, possibly by regulating the ERK signaling pathway. Our study broadens the understanding of adipogenic differentiation and provides potential therapeutic targets for the treatment of obesity.

## Supplementary Information

**Additional file 1: Fig. S1.** Differentiating OP9 and ASC cells treated with adipogenic differentiation medium. Before adipogenic differentiation, OP9 and ASC cells were incubated with 1 mM rosiglitazone to induce adipogenic differentiation. **A** Oil Red O staining; scale bar is 200 μm. **B** RT–qPCR was performed to assess the expression of the adipogenic factors *PPARγ*, *FABP4*, *FASN*, and *C/EBPα*. Values are the mean ± SEM (*n* = 3). ***P < 0.001 versus control

**Additional file 2: Fig. S2.** Slc25a5 inhibition prevents adipogenic differentiation. Before adipogenic differentiation, OP9 cells were transfected with Slc25a5 siRNA, and 24 h later, the cells were incubated with 1 mM rosiglitazone to induce adipogenic differentiation. **A** Oil Red O staining; scale bar is 200 μm (left) and 250 μm (right). **B** RT–qPCR was performed to assess the expression of the adipogenic factors *PPARγ*, *FABP4*, *FASN*, and *C/EBPα*. **C** TG levels were measured. Values are the mean ± SEM (*n* = 3). **P < 0.01 versus control

## Data Availability

The data and materials in the current study are available from the corresponding author on reasonable request.

## Abbreviations

ASCs: Adipose-derived stem cells
CCAAT: γ-Cytidine–cytidine–adenosine–adenosine–thymidine
c/EBP-α: Enhancer-binding protein-α
DEGs: Differentially expressed genes
FASN: Fatty acid synthase
FABP4: Fatty acid-binding protein 4
FBS: Fetal bovine serum
FAME: Fatty acid methyl ester
GLUT4: Glucose transporter 4
LPL: Lipoprotein lipase
OPLS-DA: Orthogonal partial least-squares discrimination analysis
PPARγ: Peroxisome proliferator-activated receptor-γ
PCA: Principal component analysis
TG: Triglyceride
