# Supplementary figures and images for: Slc25a5 regulates adipogenesis by modulating ERK signaling in OP9 cells
Source: Cell Mol Biol Lett. 2022 Feb 2;27:11. doi: 10.1186/s11658-022-00314-y (PMC8903613; doi:10.1186/s11658-022-00314-y)

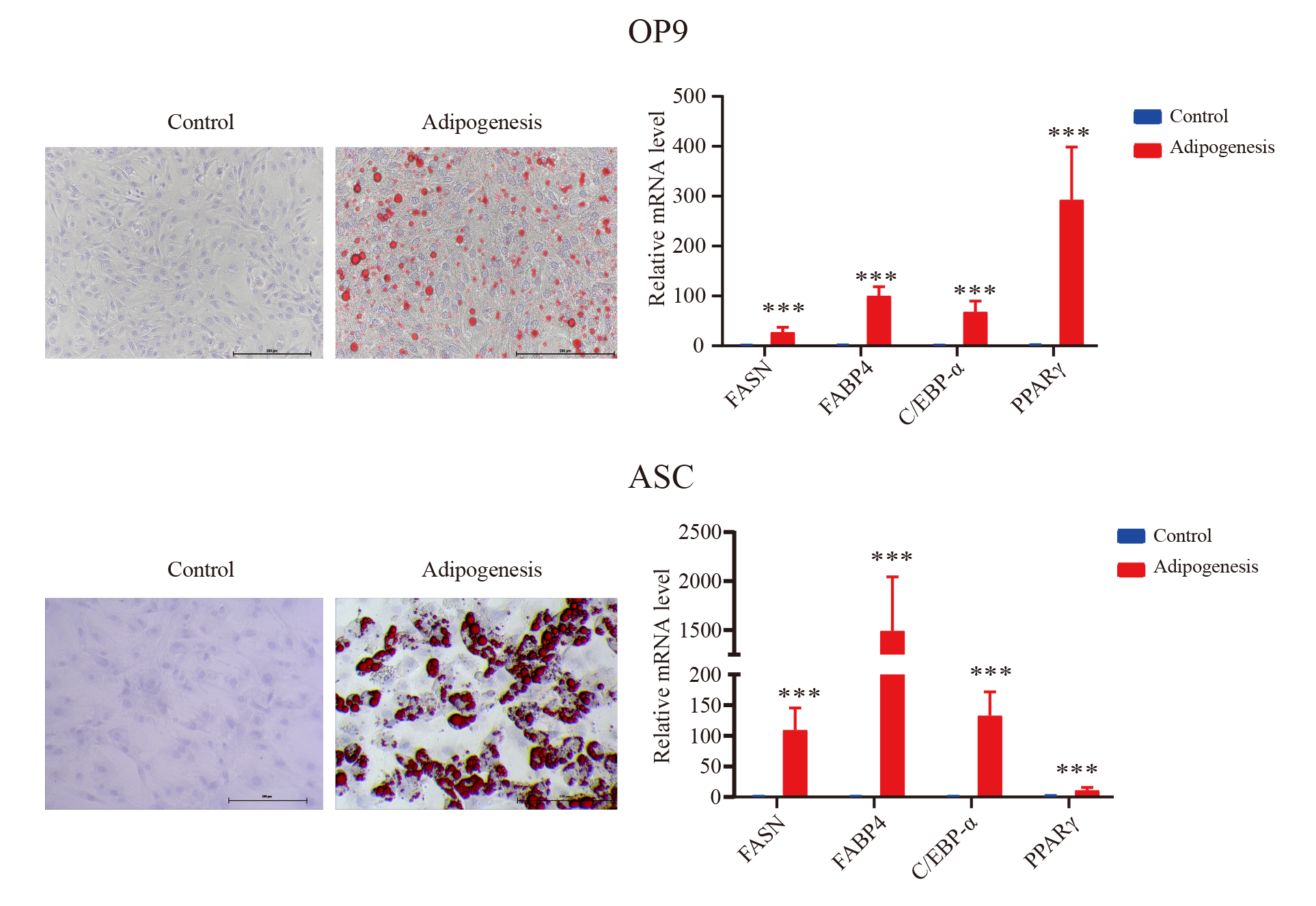

Supplement: Supplementary file 1 — Additional file 1: Fig. S1. Differentiating OP9 and ASC cells treated with adipogenic differentiation medium. Before adipogenic differentiation, OP9 and ASC cells were incubated with 1 mM rosiglitazone to induce adipogenic differentiation. A Oil Red O staining; scale bar is 200 μm. B RT–qPCR was performed to assess the expression of the adipogenic factors PPARγ, FABP4, FASN, and C/EBPα. Values are the mean ± SEM (n = 3). ***P < 0.001 versus control [file 11658_2022_314_MOESM1_ESM.tif]

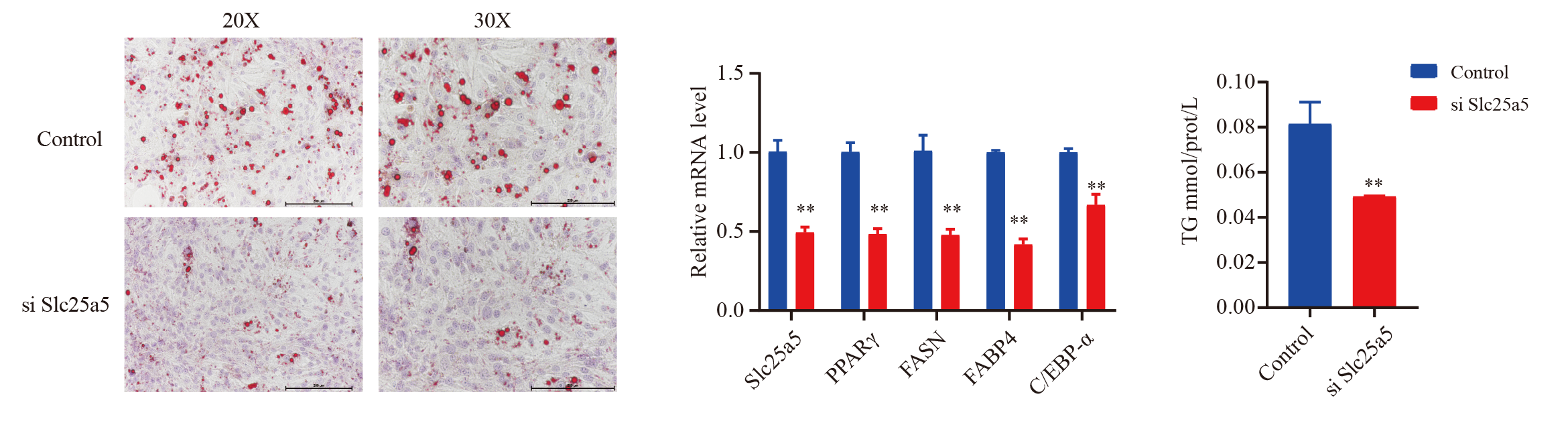

Supplement: Supplementary file 2 — Additional file 2: Fig. S2. Slc25a5 inhibition prevents adipogenic differentiation. Before adipogenic differentiation, OP9 cells were transfected with Slc25a5 siRNA, and 24 h later, the cells were incubated with 1 mM rosiglitazone to induce adipogenic differentiation. A Oil Red O staining; scale bar is 200 μm (left) and 250 μm (right). B RT–qPCR was performed to assess the expression of the adipogenic factors PPARγ, FABP4, FASN, and C/EBPα. C TG levels were measured. Values are the mean ± SEM (n = 3). **P < 0.01 versus control [file 11658_2022_314_MOESM2_ESM.tif]
